# Synergistic inter-clonal cooperation involving crosstalk, co-option and co-dependency can enhance the invasiveness of genetically distant cancer clones

**DOI:** 10.1186/s12862-023-02129-7

**Published:** 2023-05-24

**Authors:** Caroline S. Carneiro, Jorian D. Hapeman, Aurora M. Nedelcu

**Affiliations:** Department of Biology, University of New Brunswick, Fredericton, NB E3B 5A3 Canada

**Keywords:** Cooperation, Crosstalk, Co-option, Conditioned media, TGF-β1, Intra-tumour heterogeneity, Metastasis, EMT, Invasion

## Abstract

**Background:**

Despite intensive research, cancer remains a major health problem. The difficulties in treating cancer reflect the complex nature of this disease, including high levels of heterogeneity within tumours. Intra-tumour heterogeneity creates the conditions for inter-clonal competition and selection, which could result in selective sweeps and a reduction in levels of heterogeneity. However, in addition to competing, cancer clones can also cooperate with each other, and the positive effects of these interactions on the fitness of clones could actually contribute to maintaining the heterogeneity of tumours. Consequently, understanding the evolutionary mechanisms and pathways involved in such activities is of great significance for cancer treatment. This is particularly relevant for metastasis (i.e., tumor cell migration, invasion, dispersal and dissemination), which is the most lethal phase during cancer progression. To explore if and how genetically distant clones can cooperate during migration and invasion, this study used three distinct cancer cell lines with different metastatic potentials.

**Results:**

We found that (i) the conditioned media from two invasive lines (breast and lung) increased the migration and invasion potential of a poorly metastatic line (breast), and (ii) this inter-clonal cooperative interaction involved the TGF-β1 signalling pathway. Furthermore, when the less aggressive line was co-cultured with the highly metastatic breast line, the invasive potential of both lines was enhanced, and this outcome was dependent on the co-option (through TGF-β1 autocrine-paracrine signalling) of the weakly metastatic clone into expressing an enhanced malignant phenotype that benefited both clones (i.e., a “help me help you” strategy).

**Conclusions:**

Based on our findings, we propose a model in which crosstalk, co-option, and co-dependency can facilitate the evolution of synergistic cooperative interactions between genetically distant clones. Specifically, we suggest that synergistic cooperative interactions can easily emerge, regardless of the degree of overall genetic/genealogical relatedness, via crosstalk involving metastatic clones able to constitutively secrete molecules that induce and maintain their own malignant state (producer-responder clones) and clones that have the ability to respond to those signals (responder clones) and express a synergistic metastatic behaviour. Taking into account the lack of therapies that directly affect the metastatic process, interfering with such cooperative interactions during the early steps in the metastatic cascade could provide additional strategies to increase patient survival.

## Background

Despite intensive research, cancer remains the second cause of death worldwide [1]. The difficulties in curing cancer or preventing tumour progression reflect the complex nature of this disease, including high levels of heterogeneity between and within tumours. In particular, increased intra-tumour heterogeneity is generally linked to poor prognosis and poses a direct problem to therapies as drug resistance will ultimately evolve [2, 3]. According to the clonal evolution model of cancer, intra-tumour heterogeneity creates the conditions for inter-clonal competition and selection, which could result in selective sweeps and a general reduction in levels of heterogeneity [4]. However, it has been suggested that in addition to competing, cancer clones could also cooperate with each other (e.g., [5]), and the positive effects of these activities on the fitness of clones can actually contribute to maintaining the heterogeneity of tumours [6]. In addition, cooperation has been linked to drug resistance [7, 8], tumour growth [9, 10] and increased metastatic potential [11].

It should be noted that the term cooperation is generally used (including in the context of cancer) to denote either (i) an adaptive social behaviour that provides a benefit to another individual and evolved at least partially due to that benefit, or (ii) an interaction that is based on activities resulting in fortuitous benefits (such as feeding upon waste products) that, nevertheless, could evolve into cooperative behaviours (see discussion in [12]). In the former context, the actor can gain indirect benefits by directing the act towards individuals that carry the cooperative gene (e.g., involving discrimination or assortment; costly/altruistic cooperation) or direct fitness benefits that outweigh the costs of performing the behaviour (e.g., through shared interests in cooperation; mutually beneficial cooperation). Similarly, in the latter context, either one or both partners can benefit (commensalism vs. mutualism). Notably, although mutually beneficial cooperation and mutualism are often used synonymously, the two terms in fact describe very different processes: mutually beneficial cooperation implies a single behaviour that affects both the actor and recipient, whereas mutualism describes the effects that each partner has on the other—as in between species (see discussion in [12]). The most common framework for cooperation among tumour cells is the “by-product mutualism” proposed by Axelrod et al. [5], in which partially or fully transformed subclones exchange diffusible factors associated with their routine activities, which might result in benefits that neither could access alone.

Metastasis—characterized by the ability of tumour cells to migrate, invade neighbouring tissues and spread to new sites within the body, is the most lethal stage during cancer progression. To migrate, tumour cells need to undergo several changes—known as the epithelial-mesenchymal transition (EMT) [13], in which cell transition from an epithelial state (with cells connected to each other and attached to the basal membrane) to a mesenchymal state (involving the loss of cell-cell connections and adhesion) followed by the acquisition of cytoplasmic extensions and migratory abilities. The classical view of metastasis envisions single cells migrating and invading neighbouring tissues, dispersing via the vascular system, and disseminating to distant locations. However, more recent findings that some of these steps (e.g., migration, invasion, dispersal) might, in fact, involve groups of cells rather than single cells allows for the possibility that cooperation can actually contribute to successful dispersal, colonization and poor prognosis [11].

In the early stages of metastasis, such cooperative interactions can involve the induction of changes in the phenotype/state of a nearby clone that increase its metastatic potential. For example, it has been suggested that aggressive cancer cells can release signals (e.g., cytokines, chemokines, growth factors, exosomes, miRNAs) that induce EMT and enhance the metastatic potential of non-metastatic clones (see examples using melanoma [14], lung [15, 16] and breast cancer cell lines [17, 18]). Additionally, prostate mesenchymal clones were found to secrete a matricellular protein that increased the invasive capacity of epithelial clones after inducing EMT [19].

Alternatively, it has been proposed that metastatic cells facilitate the dispersal of less- or non-metastatic clones by remodelling the tumour microenvironment. For instance, leader cells can enhance the invasion of follower cells through the extracellular matrix [20, 21]. In other cases, cells that have undergone EMT degrade the surrounding matrix, which then allows the non-metastatic cells to passively enter the bloodstream and establish colonies in secondary sites [22, 23]. In one case, poorly invasive melanoma cells were found to take advantage of the more invasive cells by secreting an unidentified factor that induces a switch in the mode of invasion of the invasive cells, from proteolytic-independent to MT1-MMP-dependent [23].

Overall, despite such examples of possible inter-clonal cooperative interactions affecting the metastatic potential of a tumour, the specific interactions (in terms of the outcome for both interacting clones) and mechanisms involved in these activities are not fully understood. In most reported cases, the interaction appears to benefit one clone, while the effect of the interaction on the other clone is less clear. Nevertheless, in some cases, the interaction seems to benefit both partners, but the exact mechanisms are not known.

Furthermore, the contexts facilitating the emergence and stability of such cooperative interactions during the early stages of the metastatic process are not well understood. While kinship could favour cooperation among related subclones, the increased levels of genetic heterogeneity in advanced tumours might require additional explanations (outside the general framework of “by-product mutualism”; [5]). Furthermore, a better understanding of the general processes and mechanisms involved in initiating and maintaining cooperative activities among cancer cells could provide new therapeutic strategies and targets to inhibit the progression of metastasis.

In this study, we (i) addressed whether genetically distant clones with different metastatic potentials can cooperate to enhance each other’s and their own invasiveness (i.e., synergistic cooperation), (ii) investigated the signalling pathway involved in such inter-clonal cooperation, and (iii) developed a model for the emergence of cooperative interactions in the absence of close genetic/genealogical relatedness. To do so, we used two molecularly and cytologically different breast cancer cell lines that reflect the most common breast cancer cell types—basal (MDA-MB-231; highly aggressive) and luminal (MCF7; less aggressive) as well as a highly metastatic non-small cell lung cancer cell line (derived from NCI-H2122). To simulate a heterogeneous tumour in which different clones can affect each other through diffusible factors (crosstalk), we either subjected each line to the conditioned medium of the other line or grew the two cell lines in co-culture systems (as separate groups sharing the medium or as mixed populations in direct contact). To evaluate their metastatic behaviour, we assessed morphological changes associated with EMT and investigated the capacity of cells to migrate and invade. Based on our findings, we propose a model in which crosstalk, co-option, and co-dependency can facilitate the evolution of synergistic cooperative interactions between genetically distant clones.

## Results

### MDA-MB-231 induces an EMT-like change in MCF7

**Fig. 1:**
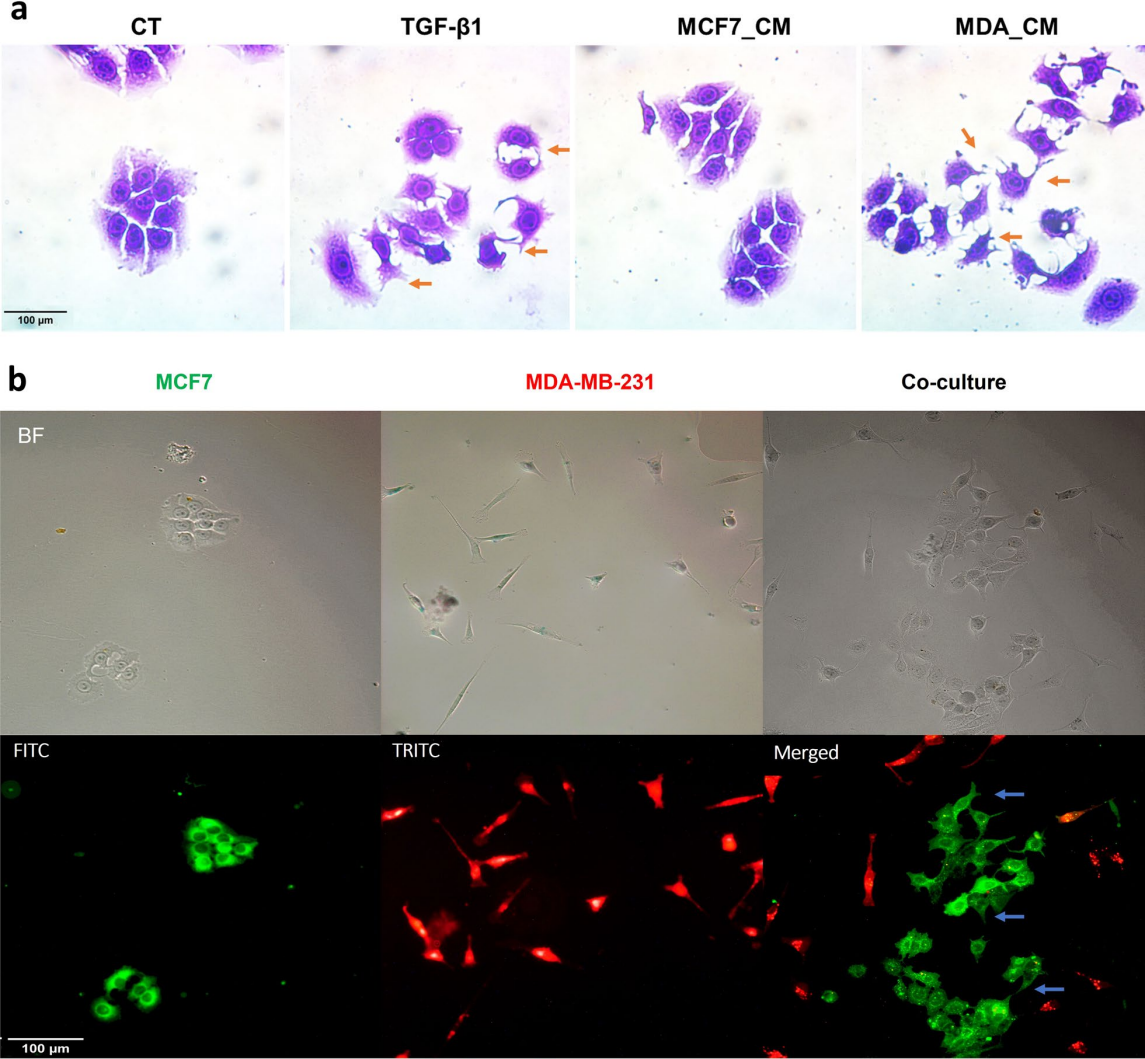
MDA induced a mesenchymal-like morphology in MCF7. **a** MCF7 cells were treated with MCF7_CM or MDA_CM and TGF-β1 (as positive control) for 72 h; cells were fixed with ethanol and stained with 0.2% crystal violet (as described in [25] and [26]). **b** MCF7 cells were cultured in direct contact with MDA cells for 72 h. Cells were stained with DiO and DiD, respectively, and fixed with paraformaldehyde 2% and then examined in bright field (BF; top row) and with fluorescence microscopy using FITC or TRITC filters (bottom row). Arrows indicate cell extensions

To address whether the highly metastatic clone, MDA-MB-231 (abbreviated as MDA from herein on), can induce an EMT-like change in the less metastatic clone, MCF7, we either subjected MCF7 to the conditioned medium (CM) collected from MDA or co-cultured the two cell lines (as a mixture, in a 1:1 ratio). We found that in the presence of MDA or its conditioned medium, MCF7 cells acquired a mesenchymal-like morphology (Fig. 1a and b). Specifically, cell extensions associated with an EMT were observed in MCF7 cultures after 72 h of treatment with MDA conditioned media (MDA_CM) or exogenous Transforming Growth Factor-β1 (TGF-β1) as a positive control (TGF-β1 is a known inducer of EMT [24])—but not when exposed to their own CM (MCF7_CM) (Fig. 1a). Similar extensions were also developed by MCF7 cells when co-cultured with MDA cells for 72 h; on the other hand, the morphology of MDA did not change when in the presence of MCF7 (Fig. 1b).

### MDA-MB-231 increases the migration potential of MCF7

**Fig. 2:**
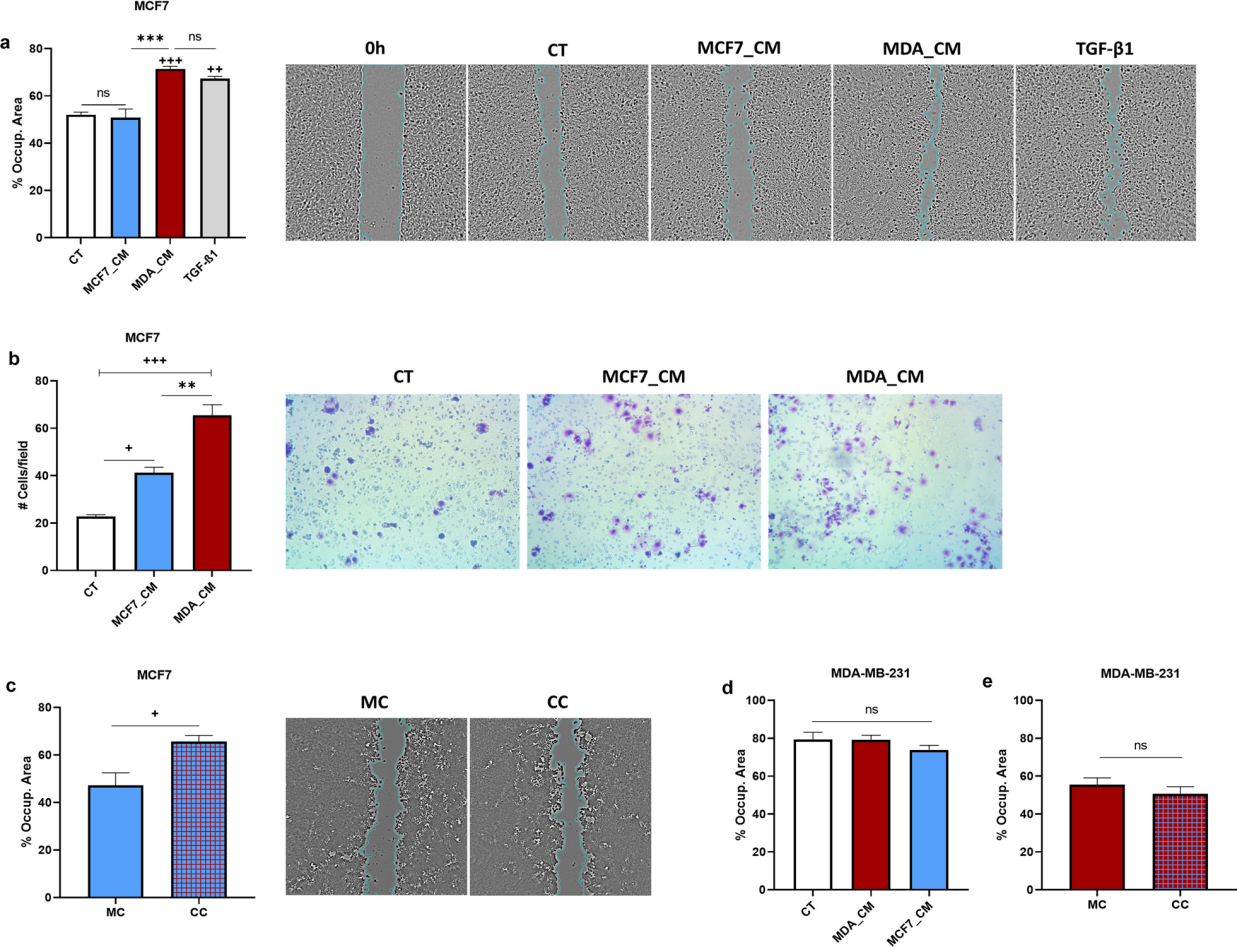
MDA enhanced the migration of MCF7. The migration of MCF7 was assessed in the presence of the CM collected from MCF7 (MCF7_CM) or MDA (MDA_CM)—using **a** wound healing assays for 48 h or **b** transwell assays for 72 h, as well as **c** in monoculture (MC) and co-culture (CC) with MDA for 72 h—using wound-healing assays. MDA migration (using transwell assays) was also assessed after 24 h. **d** in the presence of MCF7_CM or **e** in co-culture with MCF7. Y-axis indicates the percentage of the occupied area (**a**, **c**, **d** and **e**) or the number of cells per field **b**. Representative images of the gap at the end of the wound healing assays (**a**, **c**) and of the migrated cells (dark purple patches) through the transwell inserts **b** are also shown. TGF-β1 (10 ng/ml) was used as a positive control. Error bars indicate SEM (n = 3). + signs denote differences relative to control (CT), and * signs refer to differences between treatments. ns = not significant; +/*, ++/**, +++/*** denote p < 0.05, p < 0.01, and p < 0.001, respectively

To address whether the observed EMT-like changes induced by MDA are associated with an enhanced ability to migrate, we assessed the migration potential of MCF7 in the presence of the conditioned medium from MDA (using both wound-healing and transwell assays) as well as in co-culture with MDA (wound-healing assay). Overall, the migration of MCF7 increased in all contexts. Specifically, the conditioned medium from MDA significantly increased the migration of MCF7 (compared to the effect induced by its own CM) to a level similar to that promoted by TGF-β1 (Fig. 2a and b). Note that the conditioned medium from MCF7 also had a small effect on the migration of MCF7 in the transwell (but not the wound healing) assay. This can be due to a variety of factors, including the increased exposure time (72 vs. 48 h) and subsequent nutrient depletion as well as the added chemotactic component in the transwell assay (i.e., cells are attracted and respond through directional migration). The migration of MCF7 was also significantly increased when MCF7 and MDA cells were co-cultured (in separate patches in the same well) (Fig. 2c). On the other hand, the migration of MDA was not affected in the presence of MCF7_CM or when in co-culture with MCF7 (Fig. 2d and e).

### TGF-β1 is involved in the MDA-induced migration of MCF7

**Fig. 3:**
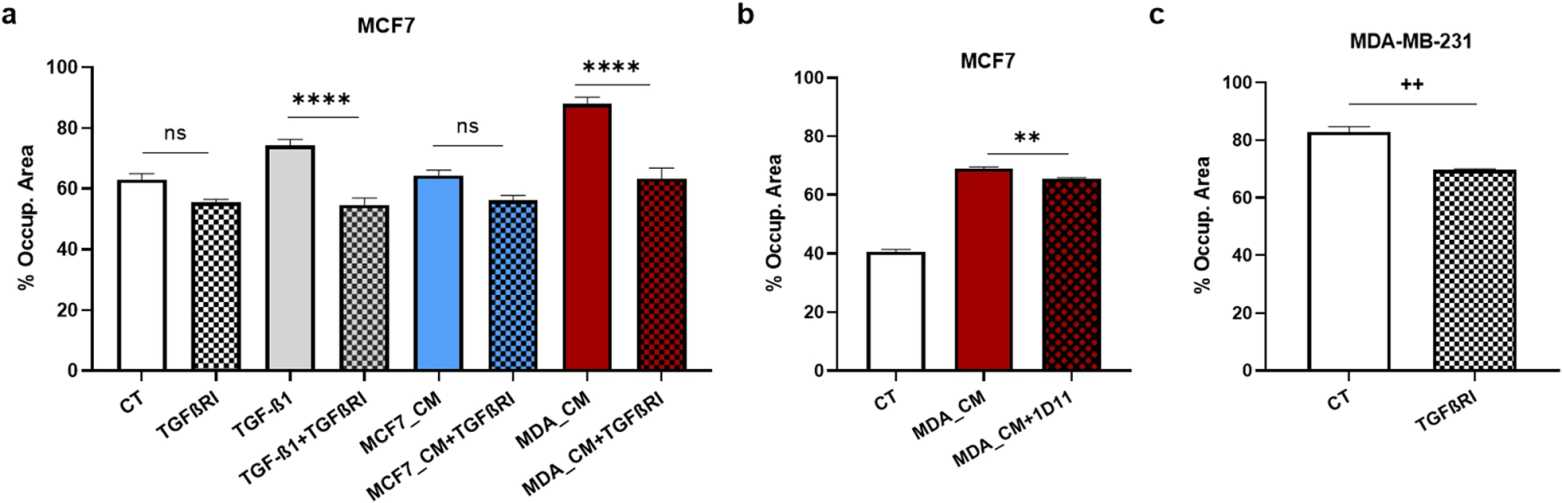
TGF-β1 is responsible for the MDA-induced enhanced migration of MCF7. **a** The effect of a TGF-β receptor I inhibitor (TGFβRI; 10 µM) on the migration of MCF7 control (CT) cultures as well as cultures exposed to either TGF-β1 (as a positive control), MCF7_CM or MDA_CM. **b** The effect of MDA_CM neutralized with antibodies (1D11) against TGF-β 1, 2, and 3 (CM + 1D11; 2 µg/ml) on the migration of MCF7 (wound-healing assay; 72 h). **c** The effect of a TGF-β receptor I inhibitor on the migration of MDA (wound-healing assay; 24 h). Error bars indicate SEM (n = 3). + refers to control (CT), and * refers to treatments. ns = not significant; ++/** denote p < 0.01; **** denotes p < 0.0001

As MDA is known to secrete TGF-β1 [27, 28], we tested whether this growth factor is responsible for the increased migration of MCF7 when in the presence of MDA. To do so, we used either a TGF-β receptor I inhibitor or antibodies against TGF-β. We found that inhibiting either the TGF-β receptor I or the TGF-β1 itself significantly decreased the effect of MDA_CM on the migration of MCF7, with the TGF-β receptor I inhibitor completely blocking its effect (Fig. 3a and b). Consistent with MDA secreting and responding to its own TGF-β1 (i.e., autocrine signalling; [28]), the TGF-β receptor I inhibitor also decreased the migration of MDA (Fig. 3c). On the other hand, the TGF-β receptor I inhibitor did not affect the un-induced migration of MCF7 (Fig. 3a), suggesting that the weak constitutive MCF7 migration does not rely on TGF-β1.

**Fig. 4:**
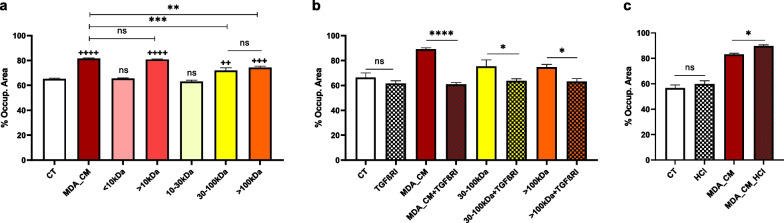
MDA_CM contains both active and inactive forms of TGF-β1. **a** Wound-healing assay was performed with MCF7 incubated with full MDA_CM and various MDA_CM fractions. **b** The effect of the TGF-β receptor I inhibitor on the ability of the full MDA_CM and its 30–100 kDa and > 100 kDa fractions to induce migration in MCF7. **c** The effect of activated MDA_CM on the migration of MCF7 in the absence (control–CT) and presence of MDA_CM; to activate TGF-β1 latent forms, pH was lowered to 4.5 with HCl and then neutralized with NaOH (wound-healing assay; 72 h). Y axis: Percentage of the occupied area. Error bars indicate SEM (n = 3). + refers to control (CT), and * refers to treatments. ns = not significant; * denotes p < 0.05; ++/**, +++/***, ++++/**** denote p < 0.01, p < 0.001, and p < 0.0001, respectively

To further confirm that TGF-β1 is involved in the effect of MDA_CM on MCF7, we fractionated the CM based on molecular weight and exposed MCF7 to the various fractions. We found that the most effective fractions were those containing molecules between 30 and 100 kDa, and above 100 kDa (Fig. 4a), which is consistent with the size of the various TGF-β1 forms—including active forms around 25 kDa and 50 kDa [29] and latent forms of ~ 95 kDa and ~ 220 kDa [29, 30]. Furthermore, the activity of these fractions was decreased by the addition of the TGF-β receptor I inhibitor (Fig. 4b). Also, as acidification can activate latent forms of TGF-β1 [31], we lowered the pH of the MDA_CM to 4.5 and observed a slight increase in its migratory effect on MCF7 (Fig. 4c).

### TGF-β1 mediates crosstalk between cancer cell types of very different origins

**Fig. 5:**
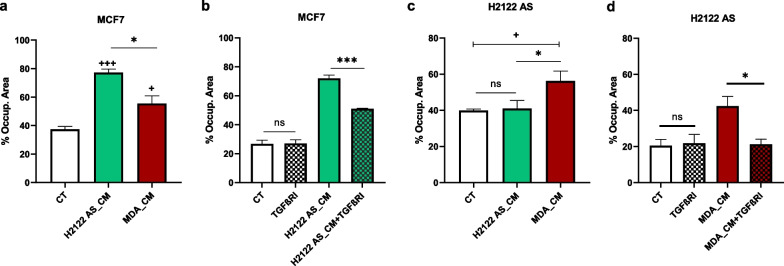
Breast and lung cancer cell lines can increase each other’s migration potential. **a** The effect of CM collected from MDA or H2122 AS on the migration of MCF7. **b** The effect of the TGF-β receptor I inhibitor (TGFβRI) on the MCF7 migration induced by H2122AS_CM (wound-healing assay; 48 h). **c** The effect of MDA_CM on the migration of H2122 AS; **d** The effect of the TGF-β receptor I inhibitor on the H2122 AS migration induced by MDA_CM (wound-healing assay; 48 h). Y axis: Percentage of the occupied area. CT–Control/Constitutive migration. Error bars indicate SEM (n = 3). + refers to control (CT), and * refers to treatments. ns = not significant; + /* and +++/*** denote p < 0.05 and p < 0.001, respectively

To explore the possibility that these findings were not specific to interactions between breast cancer cell types, we used a lung cancer cell line – H2122 AS, in conjunction with either MCF7 or MDA. First, we tested the effect of the conditioned medium from H2122 AS on the migration abilities of MCF7. We found that the inducing effect of H2122AS_CM on MCF7 migration was even higher than that of the MDA_CM (Fig. 5a). Consistent with this effect involving TGF-β1, the migration of MCF7 decreased (but was not entirely blocked) in the presence of the TGF-β receptor I inhibitor (Fig. 5b). Second, we tested whether the MDA_CM was also able to affect the H2122 AS, and found a significant increase in its migration potential (Fig. 5c). Again, consistent with this effect involving TGF-β1, the receptor inhibitor fully blocked the effect of the MDA_CM on H2122 AS migration (Fig. 5d). However, in contrast to MDA, the TGF-β receptor I inhibitor (at the same concentration) did not affect the constitutive migration of H2122 AS (Figs. 3c and 5d).

### The invasion of both MDA and MCF7 increases when in co-culture

**Fig. 6:**
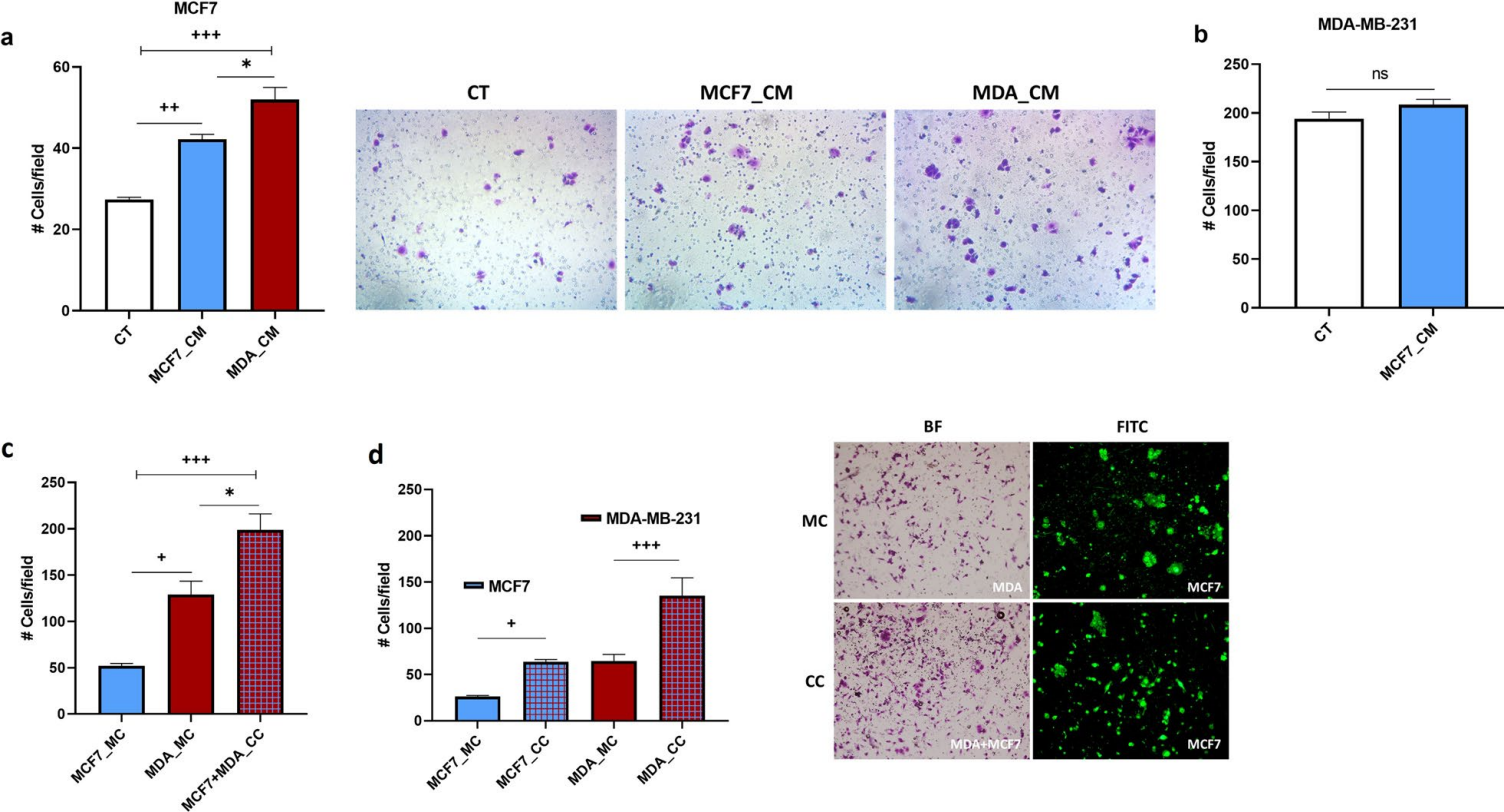
The invasive capabilities of MCF7 and MDA in the presence of CM and in co-culture. **a** MCF7 was treated with CM from either MCF7 or MDA, and **b** MDA was treated with CM from MCF7, and the numbers of invaded cells were assessed after 72 h using a transwell assay (CT – untreated control cells; constitutive invasion); representative images are also shown (dark purple patches indicate invaded cells). **c** The same total number of cells in monoculture (MC; MCF7 or MDA) and co-culture (CC; 1:1 ratio) were allowed to invade for 72 h (transwell assay), and the total number of invaded cells was assessed. **d** Comparison between the numbers of invaded cells when in monoculture (MC) and co-culture (CC) for both MCF7 and MDA; the numbers for MCF7_MC and MDA_MC represent 50% the total invaded cells in monoculture, to allow direct comparisons with the number of invaded cells of each line when in co-culture (MCF7_CC and MDA_CC) as the co-culture contained only 50% of the cells in each monoculture. To assess the total number of invaded cells, membranes were stained with 0.2% crystal violet. In co-cultures, MCF7 was stained with DiO, and the number of MDA cells was calculated by subtracting the fluorescent MCF7 cells from the total number of cells. Y axis: Number of cells per field; error bars indicate SEM (n = 3). + refers to control (CT) or monocultures, and * refers to comparisons between treatments. ns = not significant; +/* denote p < 0.05; ++ and +++ denote p < 0.01 and < 0.001, respectively

To address whether the presence of MDA can also enhance the invasive potential of MCF7, we first assessed the number of invaded MCF7 cells in the presence of MDA_CM. We found that MCF7 invaded significantly more when exposed to MDA_CM (relative to both control cells and cells exposed to their own CM; Fig. 6a). In contrast, the MCF7 conditioned media did not affect the invasion of MDA (Fig. 6b). To further evaluate the interaction between the two cell types, we mixed fluorescently labelled MCF7 and unlabelled MDA cells in a 1:1 ratio and allowed them to invade in a co-culture transwell assay. As controls, we used monocultures of each line containing the same number of cells as the total number in the co-culture (i.e., both mono and co-cultures were initiated with the same number of cells). We found that the overall number of invaded cells in the co-culture was higher than in either of the two monocultures, indicating that this mixed population had a higher overall invasion potential relative to either of the two corresponding single-clone populations of the same size (Fig. 6c). Then, to assess the direct effects of the interaction on the invasion of each line, we compared the numbers of invaded cells in co-culture vs. monoculture, for each line (Fig. 6d). We found that the number of invaded MCF7 cells more than doubled when MCF7 and MDA were co-cultured, relative to the same number of MCF7 cells in monoculture. Nevertheless, surprisingly, the number of invaded MDA cells also increased in the presence of MCF7 cells (to almost double, relative to when in monoculture) (Fig. 6d), suggesting that MDA also benefited from this interaction.

### Cooperative invasion is dependent on the ability of MCF7 to respond to TGF-β1

**Fig. 7:**
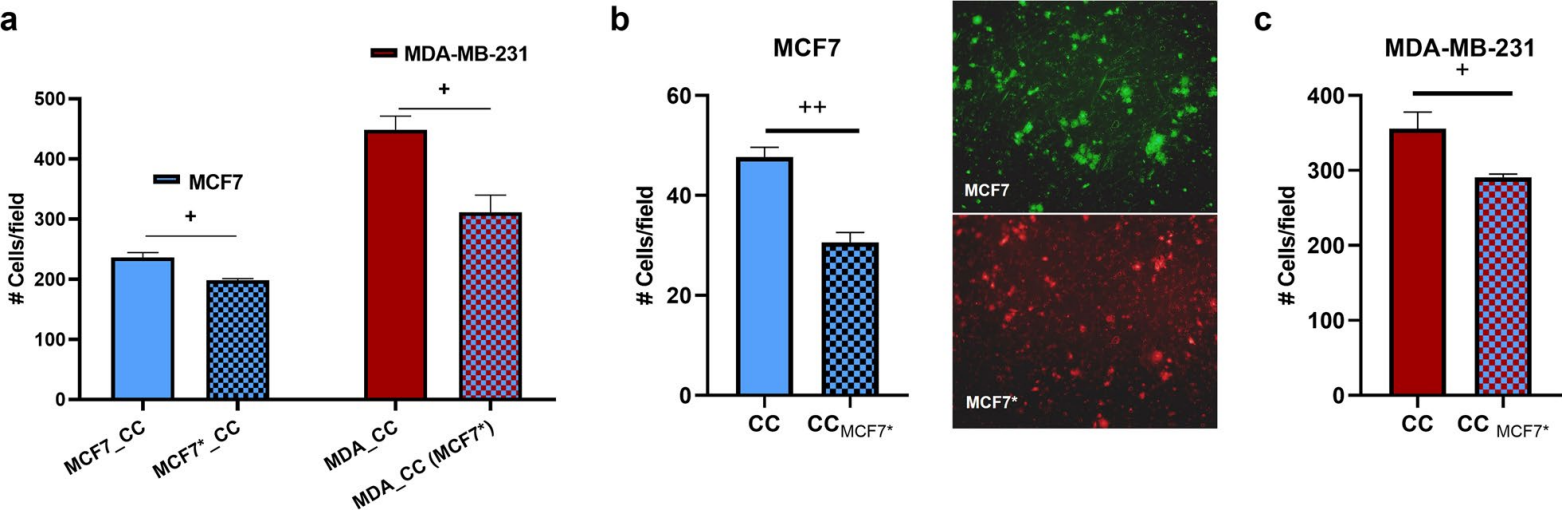
The role of the TGF-β1 receptor during cooperative invasion. **a** Comparison between the numbers of invaded cells in co-cultures (1:1 ratio) involving either receptor-positive MCF7 cells (MCF7_CC and MDA_CC) or MCF7 cells pretreated with the TGFβRI inhibitor (MCF7*_CC and MDA_CC(MCF7*). **b** Comparison between the numbers of invaded receptor-positive MCF7 cells (CC) and receptor-inhibited MCF7 cells (CC_MCF7*_) when in co-culture with MDA (1:1:2 ratio). **c** The number of invaded MDA cells when in co-culture with only receptor-positive MCF7 cells (CC; 1:1 ratio) versus a combination of receptor-positive and receptor-inhibited cells (CC_MCF7*_; 1:1:2 ratio). To assess the number of invaded cells in co-cultures, MCF7 was stained with DiO or DiD, and the number of MDA cells was calculated by subtracting the fluorescent MCF7 cells from the total number of cells stained with crystal violet. Y axis: Number of cells per field; error bars indicate SEM (n = 3). + refers to control co-culture (CC). + and +++ denote p < 0.05 and p < 0.01, respectively

To test whether the increased invasive ability of MDA was dependent on the acquired invasive phenotype of MCF7, we suppressed the ability of MCF7 to respond to the TGF-β1 released by MDA by pre-incubating the cells (for 1 h) with the TGFβRI inhibitor prior to co-culturing the two lines. We found that when MCF7 was pretreated with the inhibitor, the invasive abilities of both lines were lower relative to control co-cultures (Fig. 7a), indicating that the invasive benefit of MDA is dependent on the MCF7 being able to respond to the TGF-β1 released by MDA. In addition, to confirm that this ability does provide an invasion benefit when in a heterogeneous tumour we co-cultured both receptor-positive and receptor-inhibited MCF7 in the presence of MDA (1:1:2 ratio). As expected, the invasion of receptor-positive MCF7 cells exceeded that of receptor-inhibited MCF7 cells, indicating a competitive advantage for the former when in the presence of MDA (Fig. 7b). Also, MDA showed an increased invasion in the presence of only receptor-positive cells MCF7 relative to a mix of the two MCF7 populations (Fig. 7c).

## Discussion

In this study, we set out to fully investigate how clones with different metastatic potentials interact, with respect to both the effect of the interaction on each clone as well as the mechanisms involved. For instance, in terms of benefits, several scenarios can be envisioned: (i) the less metastatic clone benefits, with no cost or benefit to the highly metastatic clone (passive benefit; commensalism); (ii) the highly metastatic clone can take advantage of the less aggressive clone to increase its own invasion potential without a benefit to the latter (recruitment); (iii) the highly metastatic clone can co-opt the less aggressive clone into expressing a metastatic behaviour that will increase the invasiveness of both clones (synergistic cooperation). In terms of mechanisms, the highly metastatic clone can employ specific signalling molecules to induce a change in the less metastatic clone (i.e., paracrine signalling) or take advantage of the same signalling pathway involved in maintaining its own metastatic potential (i.e., autocrine signalling).

To address these potential scenarios, we chose two breast cancer cell lines with different aggressiveness levels and very different secretome profiles. MDA-MB-231 (a basal and more aggressive subtype) overexpresses more than 25 specific proteins, while MCF7 (a luminal and less aggressive subtype) secretes fifteen unique proteins [32]. Moreover, MDA releases factors that can maintain its metastatic potential (i.e., autocrine signalling) [33], including TGF-β1. In addition, to test if such interactions could be generalized to other cancer types and can involve genetically distant clones, we also used a highly metastatic non-small cell lung cancer cell line (H2122 AS). Compared to previous reports, this study not only characterized the interactions between genetically distant clones with respect to the effect on both clones but also identified the signalling pathway involved. In addition, we provide a model that can explain the emergence of cooperative interactions among genetically distant clones.

### The TGF-β1 pathway mediates the co-option of a weakly metastatic clone into expressing an increased malignant phenotype

Our data show that MDA is able to induce EMT, enhanced migration and invasion in MCF7. These findings are consistent with other studies reporting that more aggressive breast lines can enhance the metastatic potential of less metastatic lines. For instance, several aggressive breast cell lines were shown to increase the metastatic abilities of non-metastatic cells through the secretion of miR-200 packaged in extracellular vesicles [17] or through paracrine GLI activation involving Hedgehog ligands (without inducing EMT) [18]. However, in our system, we found that MDA induced EMT and increased the migration of MCF7 through soluble (i.e., not packed into exosomes) TGF-β1, and both the neutralization of TGF-β1 with antibodies and the inhibition of the TGF-β receptor I interfered with the ability of MDA to enhance the metastatic potential of MCF7. MDA is known to secrete large amounts of TGF-β1 to maintain its own metastatic potential (autocrine signalling) [34], and as expected, the inhibition of MDA’s TGF-β receptor I suppressed its migration abilities.

Interestingly, although the non-small cell lung cancer line we used is also able to secrete TGF-β1, its constitutive migration does not seem to be affected by the TGF-β receptor I inhibitor (at least not at the concentration that is effective for MDA), suggesting that its migration is not solely dependent on TGF-β1. This is consistent with a previous study reporting that inter-clonal paracrine signalling between two non-small cell lung cancer lines resulting in EMT induction involved TGF-β1 in conjunction with miRNAs [15].

Overall, our findings show that both aggressive clones (of different cancer types) secrete high levels of TGF-β1, and this released TGF-β1 can contribute to the co-option of the less metastatic clone through paracrine signalling. TGF-β1 is broadly present in the tumour microenvironment and is associated with maintaining the metastatic tumour potential in an autocrine manner [35–37]. Also, the serum levels of TGF-β1 are known to increase following tumour progression in patients with colorectal carcinoma [37], prostatic cancer [38], and breast cancer [39]; and such increase is frequently associated with poor prognosis [40]. Thus, it is likely that TGF-β1 is implicated in similar interactions in many types of cancer.

However, for the emergence of such TGFβ1-mediated positive interactions, the co-opted clone needs to express TGF-β receptors and be able to activate migration and invasion through a TGF-β1-mediated signalling pathway. Indeed, we found that MCF7 was able to respond to exogenous TGF-β1, and the inhibition of the TGF-β receptor I suppressed the effect of both TGF-β1 and the conditioned medium from MDA. Interestingly, a previous study exploring the transmission of aggressivity between breast cancer lines reported that the expression of metastatic features in MCF7 in response to conditioned medium from MDA did not involve TGF-β1 paracrine signalling [41]. Instead, the effect was attributed to the secretion of cytokines, such as G-CSF, GM-CSF, MCP-1, and IL-8. In that study, although the conditioned media from MDA increased MCF7 invasion, it only induced a partial EMT in MCF7; and MCF7 did not respond to exogenous TGF-β1 [41]. However, consistent with our findings, many other studies have reported that TGF-β1 does induce EMT (and the expression of mesenchymal markers) and migration in MCF7 [42–44]. These conflicting results might be due to the observation that MCF7 differentially expresses TGF-β receptor II depending on the cell passage number and the downregulation of Sp1 [43, 44]. In our MCF7 cell line, both TGF-β receptors I and II are constitutively expressed (our unpublished data).

### Inter-clonal synergistic cooperative interactions can increase tumour invasion potential

Positive interactions among cancer cells can be defined as unilateral (one partner passively benefits from the activity of the other; commensalism) or mutually beneficial (both partners benefit; mutualism) [5]. In this study, we have investigated the effect of inter-clonal interactions in the context of both migration and invasion. Interestingly, although MCF7 benefited from the presence of MDA in terms of increasing its own migration potential, the migration of MDA was not affected by MCF7. In other words, in terms of individual migration alone, this interaction did not appear mutually beneficial. Rather, MCF7 unilaterally increased its metastatic abilities by taking advantage of the TGF-β1 released by MDA as part of its own autocrine signalling. In theory, this interaction can result in competition between the two clones. Nevertheless, in the context of a tumour, a unilateral interaction resulting in increased metastatic potential of the recipient might still provide benefits to the actor in other fitness components. Indeed, we found that the invasive potentials of both lines were enhanced when the two lines were co-cultured in direct contact, suggesting that in vivo, such inter-clonal interactions can be mutually beneficial and synergistic.

An increase in each other’s invasiveness was previously reported when several MDA-MB-231 subclones were subjected to each other’s conditioned medium or co-cultured in vitro and in vivo, and this increase involved the release of unknown soluble factors [45]. However, in our system, the conditioned medium from non-induced MCF7 did not affect the migratory or invasive ability of MDA, suggesting that the two clones are not exchanging soluble factors. Also, MCF7 that was previously treated with an inhibitor of the TGF-β receptor I was not able to augment the migration of MDA when in co-culture. These findings argue that MCF7 cannot directly influence the behaviour of MDA, and can benefit MDA only indirectly—that is, if first exposed to the TGF-β1 secreted by MDA. In other words, the ability of MCF7 to “help” MDA is dependent on MDA first “helping” MCF7. Following this activation step, the two clones act synergistically to enhance each other’s (and their own) invasion potentials through either the shared secretion of soluble factors (e.g., proteases), taking advantage of each other’s remodelling of the extracellular matrix, or both. Notably, an increase in MMP-9 (a matrix metalloproteinase) secretion was found when MCF7 was beforehand stimulated with exogenous TGF-β1 for 24 h and co-cultured (though not in direct contact) with MDA-MB-231 [42], suggesting that TGFβ1-activated MCF7 can release invasion proteases. The collectively produced and shared proteases (i.e., public good) can then contribute to the increased invasion potential of both lines.

Similarly, in a rat mammary carcinoma cell line with two stable subtypes, an unknown soluble factor released by one subtype induced collagenase secretion by the other clone, such that collagenase could only be sufficiently secreted when both cellular types were present [46]. Also, in the melanoma system mentioned earlier [23], both protease and extracellular matrix remodelling have been found to facilitate collective invasion, though an increase in the invasion of the highly metastatic line was not reported (i.e., the interaction was not synergistic).

Interestingly, this type of inter-clonal synergistic cooperation has also been reported in the social amoeba *Dictyostelium discoideum*. Specifically, chimeric slugs of *D. discoideum* migrate farther than monoclonal slugs containing the same number of their own cells present in the mixed group (i.e., 50%), indicating that the advantages of size (i.e., increased migration potential and likelihood to reach a new food patch; [47]) resulting from “pooling” cells of different clones can drive cooperation (chimeric synergy) among unrelated clones [48]. Moreover, different species of *Dictyostelium* can also cooperate to form chimeric larger fruiting bodies, which are thought to be advantageous for overall dispersal [49].

### Co-option of autocrine signalling can facilitate cooperative interactions among genetically distant clones

**Fig. 8:**
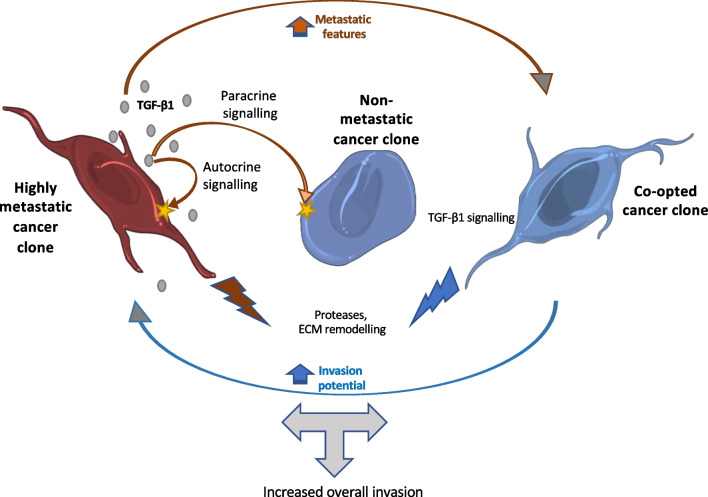
Proposed model for a TGFβ1-mediated synergistic cooperative interaction between two clones with different metastatic potentials (see text for details; ECM–extracellular matrix). Diagram created with free online Servier Medical Art at www.servier.com

Based on our findings, we propose a model (Fig. 8) whereby highly metastatic clones can co-opt (through TGF-β1 signalling) weakly or non-metastatic clones into expressing increased metastatic features, which in turn will enhance the invasion ability of the former (through released proteases and/or extracellular matrix remodelling)—i.e., a “help me help you” strategy, and ultimately result in an increase in the overall invasion potential of the tumour. Our finding that the conditioned medium from a lung cancer cell line can enhance the metastatic potential of a breast cancer line (and vice versa) indicate that this type of co-option of autocrine TGF-β1 signalling into paracrine signalling can involve genetically and phenotypically distinct clones and can take place in many types of cancer.

**Fig. 9:**
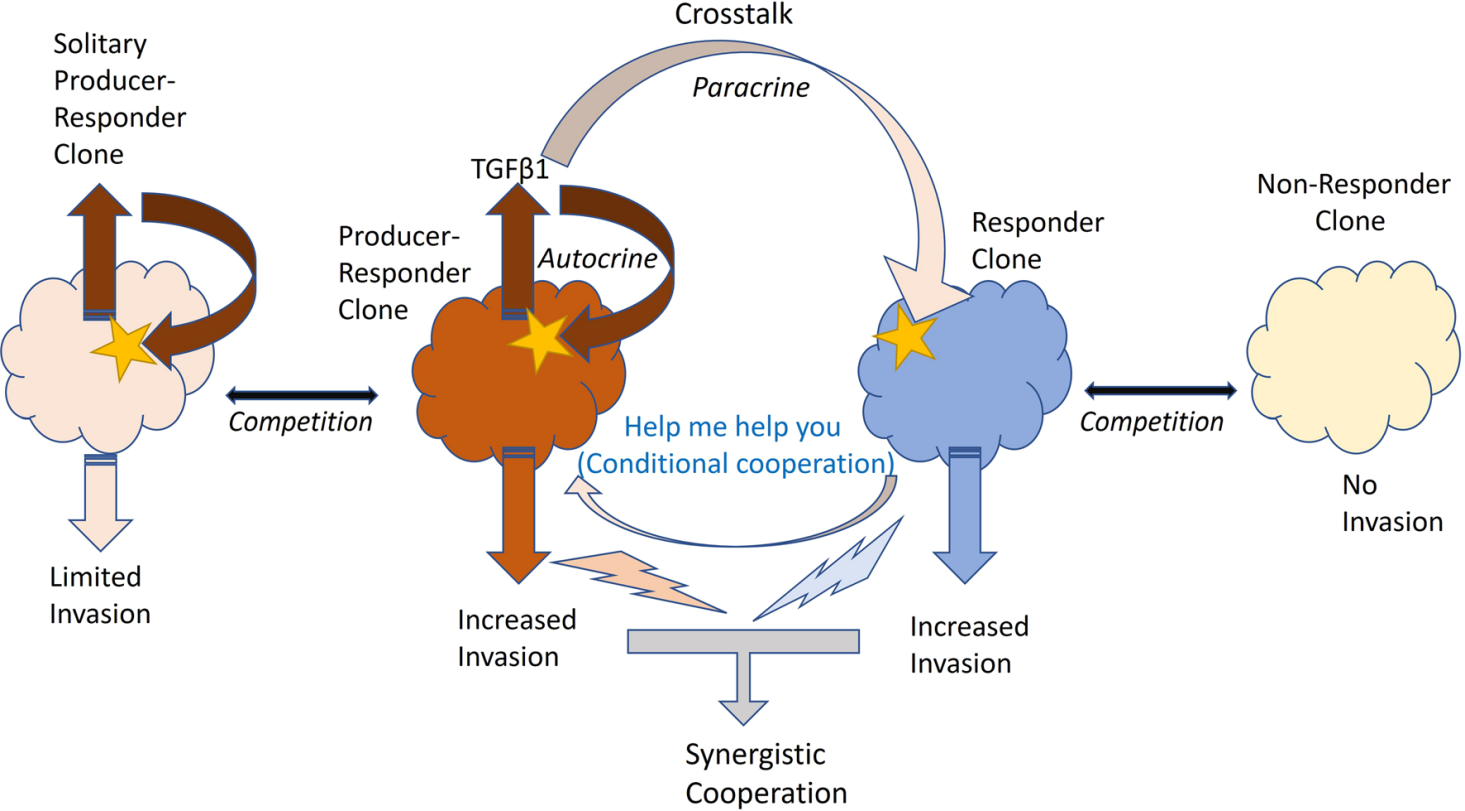
Potential interactions among various types of clones that differ in their invasion potential, highlighting the synergistic cooperation between producer-responder and responder clones through crosstalk (involving the co-option of autocrine into paracrine signalling) and co-dependency (stars denote receptors; see text for more detail)

Overall, we argue that crosstalk involving metastatic clones able to constitutively secrete signalling molecules that induce and maintain their own malignant state (producer-responder clones) and clones that express the cognate receptors to those ligands and are able to switch to a malignant phenotype (responder clones) can provide the basis for the emergence of synergistic cooperative interactions within a tumour (Fig. 9). Specifically, in a heterogeneous tumour, responder/receptor positive clones will benefit from the presence of producer-responder clones and increase their representation (through increased migration and invasion) in the nearby tissue relative to non-responder/receptor negative clones. Similarly, producer-responder clones that are found in the presence of responder clones will gain an invasion advantage relative to solitary producer-responder clones of the same size.

In this scenario, a trait (i.e., autocrine signalling) that increases individual fitness (in terms of invasion potential) in a solitary context can be co-opted into a cooperative trait if, when expressed in a heterogeneous group context, it can also increase the fitness of group members that do not express the signal but can respond to it in a similar manner (Fig. 9). If the response also benefits the producer clone, this interaction can be stable in the absence of relatedness (i.e., kin selection is not required as both clones gain direct benefits). In conditions in which the ability to invade nearby tissue provides a strong fitness advantage (as when tumour microenvironments deteriorate), such interactions can be favoured and can determine the composition of the invading population.

This is especially relevant if producer-responders are at low frequency as their invasion potential will be increased in a mixed population relative to a solitary clone of the same size (Fig. 9). Also, in later stages, such a mixed population might be favoured over a monoclonal producer-responder population as at high cell density, the increased levels of released TGF-β1 inhibit proliferation [50]. However, due to diffusion gradients and limits, these interactions will likely be dependent on cell numbers and density and be restricted to parts of the tumours, and thus could result in the coexistence of patches of cooperative and non-cooperative clones [51, 52]. Notably, basal and luminal cell clones are known to co-exist in mammary tumours (see [9, 10] and references therein).

This specific heterogeneity is also likely to affect invasion dynamics and the release of circulating tumour cell (CTC) clusters (i.e., from areas where both responder and producer-responder clones co-exist). Furthermore, the presence of both clones will reflect in the composition of CTC clusters (which are known to contain a mixture of mesenchymal and epithelial cells [53, 54]) and can also be beneficial in the subsequent steps during the metastatic process, especially the invasion and colonization of new tissues.

Although we focused on TGF-β1, the principles proposed here could apply to other signalling pathways. We suggest that such synergistic inter-clonal cooperative interactions can easily emerge between any producer-responder and responder clones that express the same cognate ligand-receptor system, regardless of the degree of overall genetic/genealogical relatedness. This model allows genetically distant clones to engage in cooperative interactions that are not initiated around public goods or exchange of products (as in most other previously proposed types of cooperation in cancer; e.g., [5]).

Furthermore, in contrast to other forms of cooperation, this cooperative system is less sensitive to inter-clonal cheating, because of the co-dependent nature of the benefit (i.e., “help me help you”). In addition, because the ability to respond to TGF-β1 provides both a cooperative benefit (e.g., shared proteases or remodelling of the tumour microenvironment) as well as a personal benefit (i.e., migration in response to TGF-β1), cheating (i.e., loss of migration by not responding to TGF-β1) can be costly; and this personal cost can stabilize the cooperative interaction, as shown in other cooperative systems [54, 55]. Lastly, although nearby non-responder clones or intra-clone cheaters could, in theory, passively benefit from this type of cooperation (if the outcome of the interaction involves the remodelling of the tumour microenvironment which could facilitate passive invasion of non-responders; [54, 55]), our data (Fig. 7b) suggest that they would still be outcompeted by the responder clones and the cooperative system will be maintained.

## Clinical implications, limitations and future research

This study underscores the importance of deciphering the nature and mechanistic basis of inter-clonal cooperative interactions that could take place during cancer progression. This is particularly relevant to metastasis—for which treatment options are limited, despite the fact that it is responsible for most cancer-related deaths [56]. Identifying the main players and signalling pathways involved in inter-clonal crosstalk during the early stages of metastasis can help develop new strategies and targets to slow down the metastatic process. One advantage of strategies targeting inter-clonal cooperation is that they would negatively affect all clones involved in a specific type of cooperative interaction, regardless of their overall genetic make-up. In this context, Metformin—a drug used to treat type 2 diabetes, could be effective in tumours producing and responding to TGF-β1 as it can inhibit both TGF-β1’s production and the response to TGF-β1 as well as inactivate the TGF-β1 itself (e.g., [57–59]).

However, as for all in vitro studies, these findings require in vivo validation. This is especially important as many signaling pathways potentially involved in inter-clonal cooperation in cancer are also associated with normal developmental and physiological processes. Therefore, these approaches should be used in conjunction with other strategies that can take advantage of specific metabolic aspects of cancer cells (e.g., sensitivity to starvation; e.g., [60] or the tumour microenvironment (e.g., hypoxia). Nevertheless, taking into account the negative impact that metastasis has on cancer prognosis and the lack of therapies that directly affect this process, interfering with the specific cooperative behaviours that tumour cells engage in should provide an additional approach to increase patient survival.

## Methods

### Cell lines and culturing conditions

The two human breast cancer cell lines used in this study, MCF7 and MDA-MB-231, were obtained from ATCC (www.atcc.org). An adherent non-small cell lung cancer line (H2122 AS) was derived from a line that grows as a mixture of adherent and suspension cells (NCI-H2122; ATCC) through selective passages of the adherent population [61]. All cell lines were grown in low/physiological glucose levels (5.5 mM; to simulate physiological glucose levels) Dulbecco’s Modified Eagle Medium (Gibco) supplemented with 10% fetal bovine serum (FBS; ATCC) and 1% Penicillin/Streptomycin (Gibco) at 37 °C with 5% CO_2_. Cells were passaged by enzymatic dissociation with 0.05% trypsin-EDTA (Gibco) when 80% confluence was reached.

### Conditioned media

To collect the conditioned media (CM), cells were grown in 5.5 mM glucose DMEM with 10% FBS until the monolayer reached around 90% confluence. Cells were then washed with PBS and maintained in DMEM with low FBS (1%) for 24 h (low FBS reduces the level of proteins and growth factors present in the conditioned medium). The collected conditioned medium was centrifuged at 1000×*g* for 10 min, and the supernatant was passed through a 0.2 μm syringe filter and stored at − 20 °C. To test the effect of CM, cells were incubated for up to 72 h with 25% CM (diluted in regular media); the CM was refreshed daily.

For some experiments, the conditioned media was also filtered using Amicon® Ultra Centrifugal Filter Units (*Millipore*) with different cut-offs (100 kDa, 30 kDa, and 10 kDa) according to the manufacturer’s instructions. The fractions were tested for their ability to induce migration.

### Reagents

Human Recombinant TGF-β1 (R&D systems; at 10 ng/ml final concentration) was used as a positive control. A TGF-β receptor I inhibitor (SB431542; EMD Millipore; 10 µM final concentration) and antibodies against TGF-β1, 2, 3 (1D11; R&D systems; at 2 µg/ml final concentration) were used to block the response to TGF-β1 and the TGF-β1 itself, respectively.

### Fluorescence staining and microscopy

For co-culture experiments, cells were stained with either DiO or DiD (Invitrogen™ Vybrant™) following the manufacturer’s instructions. The stained cells were co-cultured for 72 h and fixed with 2% paraformaldehyde. Slides were photographed at 20✕ magnification using a LEICA DM R (Epi-Fluorescence Microscope) with FITC and TRICT filters.

### Wound healing assays

To assess migration, we performed wound healing assays. Gaps within cell monolayers were achieved using cross-shaped inserts (Ibidi; www.ibidi.com). Cells were seeded at 10^5^ cells/cm^2^ in DMEM with 1% FBS (low levels of FBS inhibit cell proliferation during migration assays) and were allowed to adhere overnight. After the inserts were lifted, wells were washed with PBS, and fresh medium was added. The cells were allowed to migrate and fill in the gap while the medium was refreshed daily. For co-culture assays, each cell line was seeded in a separate insert in the same well. Once cells were attached, the inserts were removed, and DMEM with 1% FBS was added. The plates were placed on a shaker (45 rpm) to ensure the diffusion and mixing of the medium. Random fields of the gap were imaged every 24 h. At the end of the assay, when cells occupied around 80% of the gap (24 h for MDA, 48 h for H2122 AS, and 48 to 72 h for MCF7), the cell layers were fixed with 70% ethanol and stained with 0.2% crystal violet (as previous described in [25] and [26]). Gap width was measured using the Wound Healing Tool implemented in Fiji Image J. The % of the area that was occupied (Af) relative to the area at the start of the assay (Ai) was calculated ($\% occupied area=(Ai-Af)/\left(Ai\text{*}100\right)$).

### Transwell assays

Migration and invasion were also assayed using transwell inserts (Corning). 10^5^ cells were seeded in the inserts and placed in wells with media containing a chemoattractant (i.e., DMEM with 10% FBS). Cells were allowed to migrate through the 8 μm porous membrane towards the chemoattractant. After 48 h, cells that migrated to the underside of the insert were fixed with 70% ethanol and stained with 0.2% crystal violet (as previous described in [25] and [26]). Five random fields of view from the stained insert were photographed using brightfield microscopy at 10x magnification. Cells attached to the underside of the insert were counted for each field of view and averaged. The same protocol was followed to assess invasive abilities. Cells were placed onto inserts containing a layer of 1 mg/ml Matrigel (Corning), and the number of cells that invaded was assessed after 72 h. In the co-culture experiments, one cell line (MCF7) was pre-labelled with DiO or DiD, and the other (MDA) was unstained. At the end of the assay, the cells were fixed with 2% paraformaldehyde. As above, the number of invaded fluorescent cells was assessed, and all invaded cells were stained with crystal violet. The number of MDA cells that invaded was calculated by subtracting the number of invaded MCF7 cells (i.e., fluorescent) from the total number of invaded cells (stained with crystal violet).

### Statistical analyses

Each experiment included triplicate controls and treatments, and was performed several times independently. Data were expressed as mean ± SEM of the triplicates within one experiment. Unpaired, two-tailed Student’s *t*-tests or one-way ANOVA with Sidak’s test for multiple comparisons were performed to assess the statistical significance of the relevant comparisons. Data analysis was performed using Prism GraphPad 8; p ≤ 0.05 was considered statistically significant.

## Data Availability

The datasets generated and/or analysed during the current study are available in the Zenodo repository, https://zenodo.org/record/7504499#.Y7XWfdWZOUk.
